# The role of Neurotensin and its receptors in non-gastrointestinal cancers: a review

**DOI:** 10.1186/s12964-020-00569-y

**Published:** 2020-04-26

**Authors:** Stella Nikolaou, Shengyang Qiu, Francesca Fiorentino, Constantinos Simillis, Shahnawaz Rasheed, Paris Tekkis, Christos Kontovounisios

**Affiliations:** 1 Department of Colorectal Surgery, Chelsea and Westminster Hospital, NHS Foundation Trust, London, UK; 2 Department of Colorectal Surgery, Royal Marsden Hospital, London, UK; 3 Department of Surgery and Cancer, Imperial College London, Chelsea and Westminster Campus, 369 Fulham Road, London, SW10 9NH UK

**Keywords:** Neurotensin, Neurotensin receptors, Cancer

## Abstract

**Background:**

Neurotensin, originally isolated in 1973 has both endocrine and neuromodulator activity and acts through its three main receptors. Their role in promoting tumour cell proliferation, migration, DNA synthesis has been studied in a wide range of cancers. Expression of Neurotensin and its receptors has also been correlated to prognosis and prediction to treatment.

**Main body:**

The effects of NT are mediated through mitogen-activated protein kinases, epidermal growth factor receptors and phosphatidylinositol-3 kinases amongst others. This review is a comprehensive summary of the molecular pathways by which Neurotensin and its receptors act in cancer cells.

**Conclusion:**

Identifying the role of Neurotensin in the underlying molecular mechanisms in various cancers can give way to developing new agnostic drugs and personalizing treatment according to the genomic structure of various cancers.

Video abstract

## Background

Neurotensin (NT) was first detected by Carraway and Leeman in bovine hypothalamus in 1973 [1]. It is a 13-amino acid regulatory peptide present in both the central nervous system (hypothalamus and pituitary) and the gastrointestinal tract (N cells of the jejunum and ileum) [1, 2]. NT is produced from pre-pro-neurotensin, which contains the NT1–13 and neuromedin N [3]. It is released as a response to luminal fat contents and its physiological functions include increasing intestinal motility [4], increasing pancreatic and biliary secretions [2, 5] and stimulating the growth of various tissues like the gut, pancreas, adrenal gland and liver [2]. In the CNS, it is thought to inhibit dopaminergic pathways [2] and have opioid-independent analgesic properties. NT acts through 3 main receptors: Neurotensin receptor 1(NTSR1), NTSR2 and NTSR3 (or sortilin 1) [6]. The first two are seven-transmembrane G-protein coupled receptors and the latter is a single transmembrane domain sorting receptor [6].

The *NT* gene transcription is transiently expressed in fetal tissues of organs like the liver, pancreas and colon and yet is suppressed in adult tissue [7]. NT’s role in cancer is believed to stem from modifications in the *NT* gene regulation involving Ras dysregulation and changes to methylation patterns of cytosine-phosphorus-guanine (CpG) sites in the NT promoter region [6].

Among the receptors’ involvement in cancer, NTSR1 is the most studied [8]. NTSR1 is comprised of 424 amino acids and has a high affinity for NT [8]. The NT/NTSR1 complex leads to phospholipase C (PLC) activation with subsequent production of inositol triphosphate (IP3) and diacylglycerol (DAG) from membrane phospholipids [8]. Protein Kinase C (PKC) activation and intracellular calcium mobilization lead to cell proliferation, survival, migration and invasion [8].

NTSR2 is a low affinity, 410-amino acid receptor which is 64% homologous to NTSR1 [8]. NTSR2 expression has been reported in prostate cancer, glioma and chronic lymphocytic leukaemia (CLL) [6]. The exact signaling pathway involved in the role of NTSR2 is cell-dependent and currently, there is little information in its underlying mechanism of action [6, 8].

NTSR3 (sortilin 1) receptor, contrary to the other 2 receptors, is a single transmembrane domain receptor and is not specific to NT [6]. Other ligands for this receptor include lipoprotein lipase, receptor associated protein (RAP), pro-neurotrophins and sphingolipid activator protein (SAP) [6]. NTSR3’s role in cancer is largely mediated by soluble NTSR3 (sNTSR3), which is released by shedding the extracellular domain of NTSR3 [6]. sNTSR3 increases calcium concentration and induces focal adhesion kinase (FAK)/Src-dependent activation of inositol 1,4,5-triphosphate (IP3) kinase pathway, regulates cell morphology and impairs cell cohesion in colorectal cancer cell lines [6]. Sortilin has also been implicated in the tyrosine kinase and epidermal growth factor complex, exerting control on endothelial cells and angiogenesis [9].

Our team has already published a review summarizing the signaling pathway of NT in colorectal cancer and the clinical implications [10]. This review aims to explore the role of NT and its receptors in non-gastrointestinal cancers.

## Lung cancer

In the United Kingdom (UK), lung cancer is the third commonest cancer and the commonest cause of cancer death. There are two main types of lung cancer: small cell lung cancer (SCLC) and non-small cell lung cancer (NSCLC). The latter can be further subdivided into large cell lung carcinoma, squamous cell carcinoma and adenocarcinoma [11]. In the western world, the adenocarcinoma subtype of NSCLC is the commonest [11]. The 5-year survival for lung cancer is still low (10 to 20%) and the stage of the disease determines the prognosis [12]. Interestingly, within a stage, survival differs. Therefore, determining the underlying molecular pathways which drive these differences, is important. Drugs targeting epidermal growth factor receptors and their downstream signaling effectors have shown therapeutic efficacy, but eventually, there is disease progression resulting in death [13].

NT has been postulated to be one of the regulatory peptides in both SCLC [14, 15] and NSCLC [16, 17] in in-vitro studies. The postulated mechanism is that NT and NTSR1 interaction causes EGFR, HER2, and HER3 over-expression and activation of lung tumour cells [12]. In one study, blocking the NTSR1 receptor with SR48692 in human NSCLC cells, resulted in a potentiated effect of gefitinib, a tyrosine kinase inhibitor already in clinical use, in inhibiting the growth of NCI-H1299 and A549 cells [16].

NTSR1 present in the cytoplasm, as in lung adenocarcinoma is correlated with a poor prognosis, however, if it is located on the cell surface, as in lung squamous cell carcinoma, then NTSR1 has no bearing on prognosis [12]. In a clinical series of 138 stage I primary NSCLC adenocarcinomas treated only with surgery, NT was expressed in 60.4% of the cases, NTSR1 was expressed in 59.7% and both, NT and NTSR1, were expressed in 38.8% of the cases [11]. In the same study, univariate analysis showed that NTSR1 expression was associated with a worse 5-year survival rate (*p* = 0.0081) and relapse-free survival (*p* = 0.0024) [11]. In multivariate analysis, age greater than 65 years old (*p* = 0.0018) and NTSR1 expression (*p* = 0.0034), were independent negative prognostic indicators [11]. The same research group showed that high expression of NTSR1 was an independent negative prognostic marker in 389 patients with lung adenocarcinoma, stages I to III [12]. NTSR1 overexpression has also been correlated to worse sensitivity to platinum-based chemotherapy in patients with NSCLC [18]. Currently, standard chemotherapy agents include platinum-salt compound with an antimetabolite or a spindle poison [18]. Although this regime increases overall survival (OS) up to 12 months or more, patients still develop progressive disease and die [18]. Targeted therapies are now recommended in 15 to 20% of NSCLC patients who have specific mutations (e.g. in EGFR) [18]. Although these have less toxicity, most of the patients eventually develop resistance and progressive disease [18]. Resistance develops due to tumour cell plasticity and manipulation of this can improve the performance of anti-tumour drugs [18]. Wu Z et al., firstly, showed that NTSR1 over-expression is correlated with worse sensitivity to platinum-based chemotherapy agents and more tumour aggressiveness [18]. They also studied NTSR2 expression in 28 advanced stage non-squamous NSLC; staining was mainly in the cytoplasm (i.e. different to the NTSR1 distribution) and did not correlate to OS [18]. A monoclonal antibody against the long-fragment NT (LF-NT) in xenografted lung cancer cells, showed a neutralizing effect on cell growth and migration by 20–30%, as well as reduced metastasis. It also restored the cisplatin response in a dose-dependent manner without significant side effects [18].

NTSR3 or sortilin 1 has been implicated in the loading of EGFR into exosomes and sortilin depletion maintains EGFR on the cell surface, limiting EGFR endocytosis [19]. NTSR3 normally cycles between the cell membrane and the trans-Gogi network. NTSR3 binds both stimulated and unstimulated EGFR, leading to its internalization. This eventually leads to EGFR expulsion via exosomes or in receptor degradation and hence signal termination [19]. Al-Akhrass et al. found that low sortilin expression was associated with increased cellular proliferation and tumour growth, indicating that NTSR3 is a favorable prognostic marker in lung adenocarcinoma patients [19]. His team also performed immunohistochemical analysis on 78 patients with NSCLCs (grades I-III; well to poorly differentiated); sortilin expression decreased significantly with pathologic grade and was associated with poorer prognosis [19].

Currently, only EGFR-mutated tumors are eligible to receive EGFR tyrosine kinase inhibitors, which represent only 10% of all lung adenocarcinomas. Preclinical trials of erlotinib showed good response in xenografted models harbouring NT/NTSR1 but no effect if NT/NTSR1 were absent. A phase II clinical trial of Afatinib (a selective irreversible erbB family blocker) used as a third- or further-line treatment in patients with stage IV bronchial adenocarcinoma which harbor wild-type EGFR and express the Neurotensin-neurotensin receptor complex (THEN), has been withdrawn due to ‘medical reasons’ which are not further explained.

## Pancreatic cancer

**Fig. 1:**
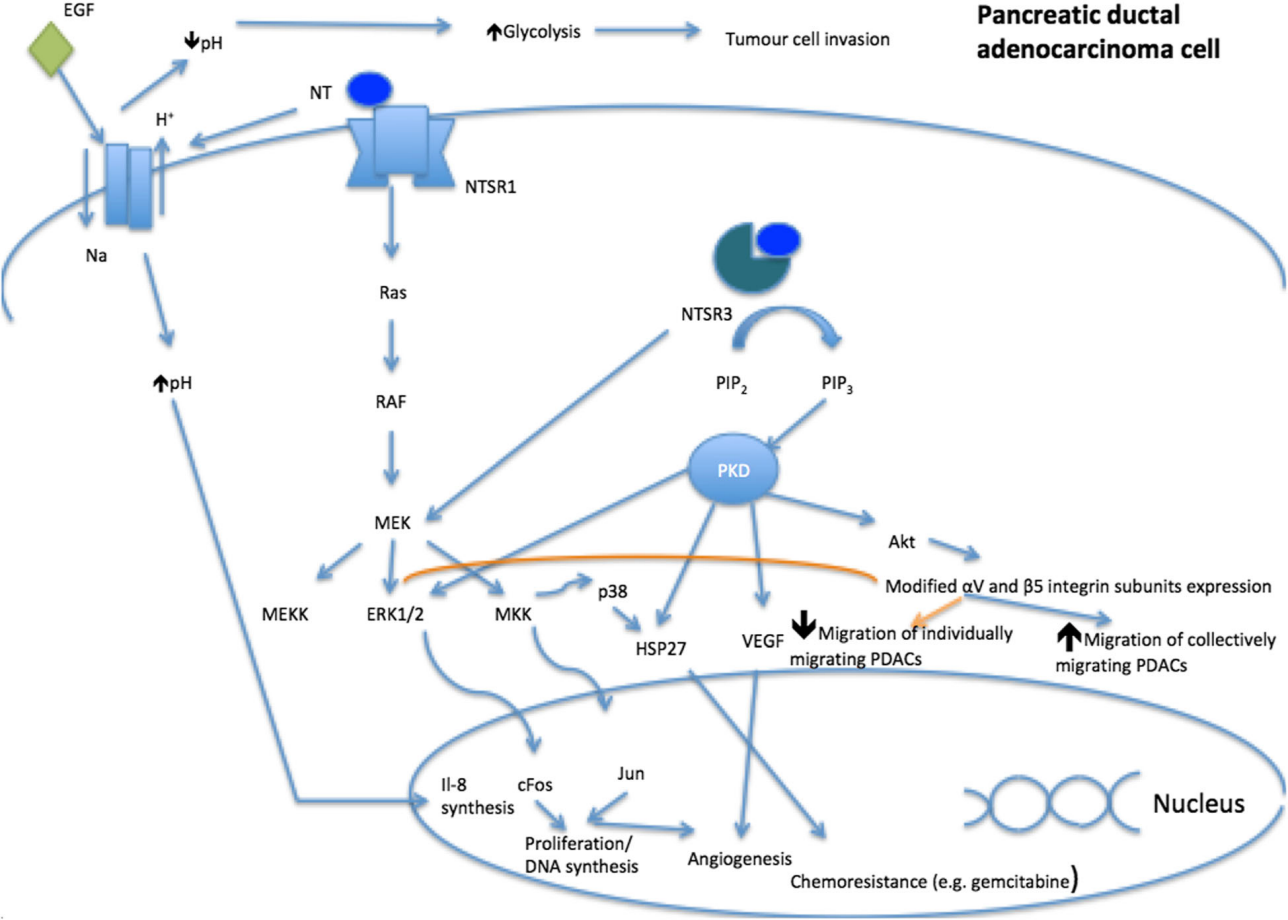
Role of NTSR1 and NTSR3 in pancreatic ductal adenocarcinoma (PDAC). ERK: extracellular signal-regulated kinases. HSP27: heat shock protein 27. IL-8: interleukin 8. MKK: MAPK kinase kinase. NT: neurotensin. NTSR: Neurotensin receptor. PIP_2:_ Phosphatidyl inositol diphosphate. PIP_3:_ Phosphatidyl inositol triphosphate. VEGF: Vascular endothelial growth factor

Pancreatic cancer is an extremely aggressive cancer with poor prognosis and this is due to the late stage of diagnosis [20]. NT has been found to increase pancreatic secretions among other physiological functions [20]. NT and its receptors play a role in the pathological growth of cancer cells, including human pancreatic cancer cells, as shown in in-vitro studies [20, 21]. Addition of NT to pancreatic cell lines stimulates DNA synthesis via protein kinase (ERK-1 and ERK-2) activation [22–25]. The combination of NT and epidermal growth factor (EGF) in PANC-1 and Mia PACA-2 cells result in a prolonged duration of ERK pathway activation leading to DNA synthesis [24]. Both NT and EGF induce extracellular acidification and intracellular alkalinization through the Na/H^+^ exchanger 1(NHE1) leading to interleukin-8 (IL-8) synthesis and promoting glycolysis leading to increased tumor cell invasion [26]. Furthermore, NT/NTSR3 complex modifies the expression of αV and β5 integrin subunits in PDACs cultured on vitronectin. This results in a reduced level of migration of collectively migrating PDACs via the PI3 kinase pathway, whilst increases the migration potential of individually migrating PDACs via the EGFR/ERK pathway [27]. Figure 1 summarises the potential pathways in carcinogenesis of NT in pancreatic cancer cells.

Pancreatic ductal adenocarcinoma overexpresses NTSR1 and NT, whilst normal pancreatic tissue does not [28, 29]. The intensity of NTSR1 increases with the stage of pancreatic cancer and metastases express NTSR1 at the same level as the primary tumour [30]. Preliminary studies showed that NTSR1 is a promising target for therapeutic and diagnostic purposes [31, 32]. Baum and colleagues tested the use of radiopharmaceutical therapy, using an NTSR1 antagonist, in 6 patients with advanced pancreatic adenocarcinoma [30]. They used ^177^Lu-3BP-227 as an intravenous agent to assess uptake and then one of the patients proceeded to have 3 further intraperitoneal doses. Tumor uptake was observed in 5 out of 6 patients and one of the patients achieved a partial remission with improved quality of life and 11-month survival despite a very poor prognosis [30]. There is currently an ongoing phase I and II trial, in evaluating ^177^Lu-3BP-227’s safety and efficacy in various cancers including pancreatic cancer [33].

## Breast cancer

Breast cancer is one of the commonest causes of cancer deaths in the western world and despite improvements in early detection, surgery and targeted therapy, one in four women still die from this disease [34]. This is mainly due to the development of metastatic disease and it is therefore important to identify new markers of progression and develop highly specific treatments targeted to metastasis to improve survival [34].

NT and NTSR1 have been implicated in breast cancer progression [8, 35] and high levels of pro NT in the blood is linked to a higher risk of breast cancer [36]. The NT/NTSR1 complex enhances tumour growth and metastasis [37].

A study by Souaze et al. showed that 34% of invasive ductal breast carcinoma (IDC) specimens express NT, 91% express NTSR1 and 30% express both NT and NTSR1 [38]. The same team also showed that NTSR1 was involved in cellular migration, invasion and induction of matrix metalloproteases-9 (MMP-9) and use of SR48692 (an NTSR1 antagonist) halted tumor growth in triple-negative cancer cells (MDA-MB-231) xenografted in nude mice [38]. Subsequent studies in triple-negative breast cancer phenotype cells (MDA-MB-231) which were xenografted in nude female mice, and were treated with both intravenous and intratumoral NT polyplex nanoparticles harboring the herpes simplex virus thymidine kinase suicide gene and ganciclovir, showed a significant growth inhibition (55–60%) (*p* < 0.001) [39].

High NTSR1 in patients with IDCs is correlated with larger tumor size, Scarff-Bloom-Richardson (SBR) histoprognostic grade 3 and number of positive lymph nodes. These patients also have worse survival compared to those with low NTSR1 expression (10-year survival rate of 66.2% versus 96.5%, *p* = 0.01) [34].

The NT/NTSR1 complex enhances tumour aggressiveness by increasing human epidermal growth factor receptor (HER) expression and activation via the release of specific EGFR, HER2 and HER3 ligands [37]. Use of lapatinib, an EGFR/HER2 tyrosine kinase inhibitor, and metformin reduced the growth of cells overexpressing NT/NTSR1 in an experimental mouse model, suggesting that NT/NTSR1 as a potential therapeutic target [37].

## Prostate cancer

Androgen treatment provides a temporary therapeutic response in prostate cancer, as eventually, a more aggressive and androgen-independent tumour tends to develop after initial therapy [40]. Discovery of molecular targets is therefore important in treating advanced prostate cancer.

The NT receptor pathway has been particularly implicated as an alternative growth pathway, especially in the absence of androgens [41, 42]. NT, binding to NTSR1, causes prostatic cancer cell mitosis through Src-, MMP- and PKC-dependent transactivation of EGFR, which ultimately stimulate the MAP-kinase pathway in a PI3K-dependent manner [43–45]. NT and its receptors have been implicated in the neuroendocrine differentiation of prostate cancers [46]. Moreover, NTSR1 blockade with the selective NTSR1 antagonist, SR48692, inhibits Neurotensin-mediated prostatic cancer growth [44, 47].

Patients treated with long-term anti-androgen therapy, tend to develop a more aggressive type which mainly consists of neuroendocrine cell clusters [48]. In malignant cell lines, NTSR1 expression was found in poorly differentiated (androgen receptor-negative) cell lines, whilst NTSR2 was found only in well-differentiated malignant cell lines. NTSR3 was found in all cell lines [40]. NTSR1 was also more highly expressed in basal-like phenotypes, which tend to lack AR, suggesting that NTSR1 supplies the mitogenic stimulus as an alternative pathway to carcinogenesis [40]. NTSR2 and NTSR3 were more highly expressed in luminal-like phenotypes [40]. Interestingly, in benign prostate tissue sections, NTSR1 was expressed in basal and luminal compartments, as well as in the stroma [40]. A more recent study, studying the NTSR1 expression in normal, benign prostatic hyperplasia (BPH), prostatic cancer and metastatic lymph nodes, there was no clear association between NTSR1 and age, PSA values, Gleason score, pathological T stage, but there was more frequent NTSR1 overexpression in metastatic lymph nodes compared to primary tumours (*p* = 0.038) [49]. The authors concluded, in this study, that this may open a new perspective in imaging and radionuclide therapy in prostate cancer [49].

Deng and colleagues in 2017 used 3 ^(64)^ Cu chelators which were conjugated to an NT analog and NTR binding affinity was evaluated using cell-binding assay, in human prostate cancer PC3 xenografts. PET/CT imaging of NTR expression showed high tumour uptake of the probes correlating with the in vitro Western blot results. The authors concluded that these agents may help identify NTR-positive lesions and predict which patients and individual tumours are likely to respond to novel interventions targeting NTSR1 [50].

Geer S and colleagues tested the uptake of Lu-177-labelled NTSR1 antagonists into a cumulative tumour dose of 1.25Gy/MBq, in prostate specific membrane antigen (PSMA) negative and NTSR1 positive prostate tumour-bearing nude mice. The tumour growth over 57 days and survival was significantly different between the treatment and control group, showing that NTSR1 could be a suitable molecular target in PSMA-negative prostate carcinoma for radiotherapy [51].

## Head and neck tumours

Head and neck squamous cell carcinomas (HNSCC) represent 6% of all cancers and their overall 5-year survival is amongst the worst compared to other major cancer types [52]. This is largely due to local recurrence and distant metastasis after standard treatment, as well as the heterogeneity of the disease [53].

A study by Shimizu and colleagues in 2008 was the first to identify a link between NT-NTSR1 complex and HNSCC [52]. After a genome-wide gene expression analysis on HNSCC specimens revealed that high mRNA expression levels of NT and NTSR1 had a worse metastasis-free survival rate [52]. In HNSCC cells, the addition of NT promoted invasion and migration, whilst knockdown of the NTSR1 had the opposite effect [52].

## Pleural mesothelioma

Malignant pleural mesothelioma (MPM) has a poor prognosis with a median survival of 8.9 months [54]. More recently, treatment with induction therapy and radical surgery has improved survival to greater than 20 months [55]. Unfortunately, only a small proportion of patients with MPM are eligible for such aggressive treatment due to late presentation, significant comorbidities and histological type [54].

In-vitro studies show that inhibition of the NT system reduced migration and collagen invasion of mesothelioma cells [54].

Thirty per cent and 77% of normal pleura express NT and NTSR1 respectively [8]. In MPM, this percentage rises to 71.1 and 90.4% for NT and NTSR1, respectively. Interestingly, NTSR1 is expressed in the cytoplasm of tumoral cells, whilst it is expressed only on the cell membrane of normal pleural cells. High NT expression is associated with poor prognosis [54].

## Glioma

Gliomas comprise 80% of all malignant brain tumours and despite the improvement in neurosurgery, radiotherapy and chemotherapy, their high recurrence rate accounts for the high mortality for these patients [56]. Gliomas are characterized by their rapid proliferation and extensive invasion, hence finding therapies to target this, would be of great interest [56].

NT is known to be present in the central nervous system and is highly expressed in the hypothalamus, median eminence, pituitary stalk, substantia nigra, locus coeruleus, raphe nuclei and brainstem structures [57]. In-vitro studies showed that NT stimulates Erk1/2 phosphorylation and is upregulated in glioma cells [58]. Both NT and NTSR1 is correlated with increasing glioma tumour grade [56]. In-vivo experiments in xenografted mice models showed that NTSR1 blockade significantly prolonged survival [56, 58].

Glioblastoma is the most aggressive brain tumour with a median overall survival of 12 to 15 months [59]. Among the gliomas, glioblastoma mutliforme (GBM) has the highest expression of NT and NTSR1 which in turn is associated with increased postoperative mortality [56]. Interestingly, different GBM cell lines express different receptors (GL261, U087MG, U-118MG and A172 lines express NTSR1; C6 line expresses NTSR2; U-373MG line express NTSR1–3) [60].

The exact mechanism of NT in GBM is not well understood. Several in-vitro studies suggest that NT acts on glioblastoma stem cells (GSC) via an IL-8/CXCL8 mechanism [61]. NT activates NTSR1 and EGFR which in turn increased the expression of IL-8 and CXCL8 in GSC, which go on to activate the CXCR1 receptor. The latter activates the STAT3 transcriptional factor. This leads to increased proliferation, migration and invasion [62].

NT also seems to have an important role in protecting glioblastoma from intrinsic apoptosis via c-Myc/LIN28/Let-7a-3p/Bcl-w axis [63]. NT increases tumour sphere formation and regulates stem-like traits via an EGFR-dependent increase of interleukin-8 (IL-8) secretion [61].

NTSR1 activation increases CDK4 and CDK6 expression via c-myc and miR-129-3p/miR/29b-1 respectively [64]. Furthermore, knockdown of NTSR1 reduces tumour invasion via the Jun/miR-494/SOCS6 axis [65].

## Liver cancer

Hepatocellular carcinoma is a primary liver malignancy and the fourth commonest cause of cancer-related death worldwide [66]. Underlying risk factors include chronic liver conditions, for example, chronic hepatitis B and C, alcohol addiction and non-alcoholic fatty liver disease [66]. Chronic inflammation is thought to be a driving factor for tumour invasion and progression, regardless of the cause [67, 68]. NT has been reported to promote cytokine release, particularly IL-8 via the MAPK and NF-kB pathways, enhancing the migration and invasion capacity of HCC cells [69]. Overexpression of both interleukin-8 and NT in HCC has been associated with portal vein invasion and worse prognosis [69, 70]. Furthermore, co-expression of NT and NTSR1, found in 50% of HCC, has also been found to be correlated with poor prognosis and alteration of the Wnt/ß-catenin pathway [71, 72].

Fibrolamellar hepatocellular carcinoma (FL-HCCs) is a rare form of HCC which tends to happen in teenagers and young adults, without underlying liver disease [73]. The underlying pathogenesis is due to an overexpression of the fusion protein DNAJ-PKAc and the PKAc kinase activity which depends on cyclic AMP (cAMP). NT and its receptors, NSTR1 and NTSR2, are a potential source of cAMP which in turn increases PKA activity [73]. In-vitro studies of cultured hepatocytes show that NT and NTSR1 act as co-mitogens on EGF signaling by promoting EGFR phosphorylation and MAPK pathway activation [73]. NT may also act in a paracrine/autocrine method, where NT binding to its GPCR, activates *NT* gene expression, propagating NT protein production [73].

## Malignant melanoma

Malignant Melanoma (MM) is a highly aggressive skin cancer with an increasing incidence worldwide [74]. Early detection and surgical excision can cure the disease, however more advanced stages are resistant to the current chemo-radiotherapy regimens, leading to a poor prognosis [75].

There has only been one study investigating the link between NT and MM. This showed that NT is highly expressed in melanoma cells(A375 and MV3 cells) but its expression in normal immortalized human keratinocyte line HaCaT is low [75]. The same study found that NT/NTSR1 activation is required for cell migration in the A375 melanoma cells. Inhibition of NTSR1 with SR48692 antagonist induced apoptosis in A375 melanoma cell line and reduced tumour growth in vivo [75]. The authors suggested that NTSR1 may be a novel drug target in melanoma, however, there are no further studies to investigate its potential.

## Leukaemias

In-vitro studies show that NTSR1 is expressed in HL-60 human myeloid leukaemia cells [76].

NT, NTSR1, NTSR2 and NTSR3 are all expressed in human B cell lines [77]. Exogenous NT promotes proliferation and apoptosis in human B cell lines via the NSTR1 activation pathway [77]. NTSR2 and NTSR3 were also overexpressed in chronic lymphocytic leukaemia (CLL) B cells [77, 78]. Farahi and colleagues postulated that NSTR3 expression could potentially be a diagnostic but not a prognostic marker in CLL [78]. He further showed that treatment with monoclonal antibodies against NSTR3 selectively killed CLL cells but not healthy peripheral blood mononuclear cells (PBMCs) [78].

## Miscellaneous

NT receptors have been found in varying degrees in Ewing’s sarcoma, meningiomas, sarcomas, astrocytomas, medulloblastomas and medullary thyroid carcinomas [79].

## Conclusion

**Fig. 2:**
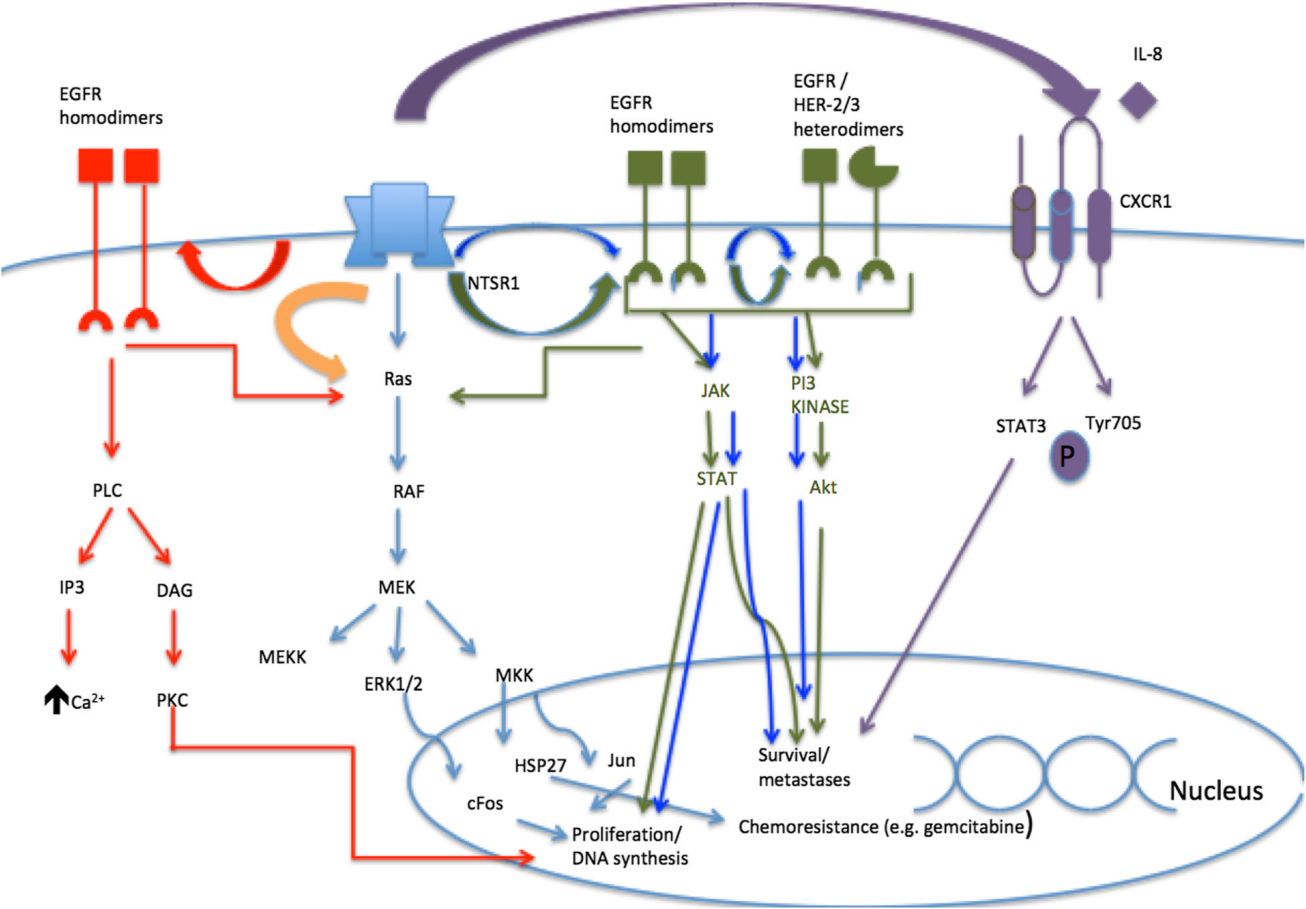
Summary of the role of NT/NTSR1 in non-gastrointestinal cancers.  Role of NT/NTSR1 in pancreatic ductal adenocarcinoma.  Role of NT/NTSR1 in prostate cancer.  Role of NT/NTSR1 in non small cell lung cancer.  Role of NT/NTSR1 in breast cancer.  Role of NT/NTSR1 in glioma.  Role of NT/NTSR1 in glioblastoma multiforme. DAG: diacylglycerol. EGFR: epidermal growth factor receptor. ERK: Extracellular signal-regulated kinases. HER: Human epidermal growth factor receptor. HSP27: Heat shock protein 27. IP3: Inositol triphosphate. PKC: Protein kinase C. MAPK: Mitogen activated protein kinase

Preliminary studies have shown NT and its receptors to promote cell proliferation, DNA synthesis, migration and angiogenesis through autocrine and paracrine effects in many cancers (Fig. 2). Their direct effect on MAPK pathway, EGFR transactivation and Protein kinase C and D pathway activation makes them an interesting target in therapeutic use in the subset of cancers which express NT and/or its receptors, making way for a promising new tumour agnostic drug. They also show potential as prognostic and predictive markers. Although there is promise in the use of these markers in animal models, further study is required to assess its applicability in clinical use (Table 1).

**Table 1:** A summary of Neurotensin (NT) and its receptors research studies

Site of cancer	In-vitro	Animal models	Prognostic/Predictive studies	Clinical trials
Lung (NSCLC)	Treatment with NT increased growth in LMN-R cells	NTSR1 blockade potentiates effect of geftinib	NTSR3 good prognostic marker	Afatinib in stage IV bronchial adenocarcinoma
			NTSR1 in cytoplasm a.w. poor prognosis	
			NTSR1 predicts worse sensitivity to platinum based treatments	
			NTSR2 expression has no correlation to OS	
Pleural mesothelioma	NTSR1 antagonist reduced cell invasion in MSTO-211H cells		High NT expression is an independent predictor of poor 5-year survival	
			NTSR1 had no impact of survival	
Pancreas	NTSR1 promotes DNA synthesis, proliferation, angiogenesis		NTSR1 increases with stage of disease	NI
	NTSR3 increases migration in individually migrating PDAC cells			
Breast		NTSR1 antagonist arrested tumour growth in xenografted MDA-MB-231 cells	High NTSR1 in patients with IDCs correlated to higher histoprognostic grade, larger tumour size and higher number of positive lymph nodes	NI
		Suicide HSVtk gene delivery by NT-polyplex nanoparticles + ganciclovir to treat MDA-MB-231 cells		
			High NTSR1 expression is associated with worse survival	
Prostate	NT/NTSR1 in PC3 cells stimulates EGFR/ERK/Akt phosphorylation and DNA synthesis which is PKC-dependent	^(64)^ Cu chelators conjugated to a NT analog was assessed as an imaging marker using PET/CT to was correlated to NTSR1-positive lesions Use of Lu-177-labelled NTSR1 antagonists in PSMA negative and NTSR1 positive prostate cancer nude mice showed reduced tumour growth over 57 days	There is more frequent NTSR1 overexpression in metastatic lymph nodes compared to primary tumours	
	NTSR1 is found in poorly differentiated (androgen receptor negative) NTSR2 was found in well-differentiated cell lines			
	NTSR3 found in all cell lines			
HNSCC	Addition of NT promoted invasion and migration. Knockdown of NTSR1 slowed invasion and migration	NI	High mRNA expression of NT and NTSR1 has a worse metastasis-free survival rate	NI
Glioma Glioblastoma multiforme (GBM)	NT stimulates ERK1/2 phosphorylation	NTSR1 blockade in xenografted nude mice increased survival	NI	NI
	Different GBM cell lines express different NT receptors	NI	NI	NI
	Studies on glioblastoma stem cells show that NT acts through the CXCR1/2/IL-8 pathway which is EGFR-dependent			

*CXCR* CXC chemokine receptor, *EGFR* epidermal growth factor receptor, *ERK* extracellular signal-regulated kinase, *IDC* invasive ductal carcinoma (breast), *IL-8* interleukin 8, *MDA-MB-231* triple negative breast cancer cells, *NTSR* neurotensin receptor, *NI* not investigated, *OS* overall survival, *PC3* cell line from bone metastasis of prostate cancer patient, *PDAC* pancreatic ductal adenocarcinoma cells, *PKC* protein kinase C, *PSMA* prostate specific antigen

## Data Availability

Not applicable.

## Abbreviations

AR: Androgen receptor
BPH: Benign prostatic hyperplasia
CDK: Cyclin-dependent kinase
CLL: Chronic lymphocytic leukaemia
CpG: Cytosine-phosphorus-guanine
Cu: Copper
CXCL8: Chemokine (C-X-C) ligand
CXCR: Chemokine receptor
DAG: Diacylglycerol
DNA: Deoxyribonucleic acid
EGFR: Epidermal growth factor
EMT: Epithelial-to-mesenchymal transition
ERK: Extracellular signal-regulated kinases
GBM: Glioblastoma multiforme
GSC: Glioblastoma stem cells
IL-8: Interleukin-8
IDC: Invasive ductal carcinoma
FAK: Focal Adhesion kinase
HER: Human epidermal growth factor receptor
HNSCC: Head and neck squamous cell carcinomas
IP3: Inositol 1,4,5- triphosphate
MAPK: Mitogen-activated protein kinase
MDA-MB-231: Is a highly aggressive triple negative breast cancer cell line
MMP: Matric metalloproteinases
MPM: Malignant pleural mesothelioma
OS: Overall survival
PKC: Protein Kinase C
PLC: Phospholipase C
LF-NT: Long fragment NT
RAP: Receptor-associated protein
NHE1: Sodium/ Hydrogen exchanger 1
NSCLC: Non-small cell lung carcinoma
NT: Neurotensin
NTSR1: Neurotensin receptor 1
NTSR2: Neurotensin receptor 2
NTSR3: Neurotensin receptor 3
PANC: Pancreatic cancer cell line
PC3: Prostate cancer cell line
PDAC: Pancreatic ductal adenocarcinoma
PSA: Prostate specific antigen
PSMA: Prostate specific membrane antigen
SAP: Sphingolipid activator protein
SBR: Scarff-Bloom-Richardson grading score
SCLC: Small cell lung carcinoma
sNTSR: Soluble Neurotensin receptor
THEN: The Neurotensin-neurotensin receptor complex
